# Scaled transverse translation by planar optical elements for sub-pixel sampling and remote super-resolution imaging

**DOI:** 10.1515/nanoph-2024-0600

**Published:** 2025-02-10

**Authors:** Qi Zhang, Xin Xu, Yinghui Guo, Yuran Lu, Qiong He, Mingbo Pu, Xiaoyin Li, Mingfeng Xu, Fei Zhang, Xiangang Luo

**Affiliations:** State Key Laboratory of Optical Technologies on Nano-Fabrication and Micro-Engineering, 74709 Institute of Optics and Electronics, Chinese Academy of Sciences, Chengdu 610209, China; National Key Laboratory of Optical Filed Manipulation Science and Technology, Institute of Optics and Electronics, Chinese Academy of Sciences, Chengdu 610209, China; Research Center on Vector Optical Fields, Institute of Optics and Electronics, Chinese Academy of Sciences, Chengdu 610209, China; School of Optoelectronics, University of Chinese Academy of Sciences, Beijing 100049, China; Sichuan Provincial Engineering Research Center of Digital Materials, Chengdu 610299, China; Tianfu Xinglong Lake Laboratory, Chengdu 610299, China

**Keywords:** quadratic-phase metasurface, planar optical element, sub-pixel sampling, super-resolution imaging, fourier ptychography imaging

## Abstract

High resolution imaging represents a relentless pursuit within the field of optical system. Multi-frame super-resolution (SR) is an effective method for enhancing sampling density, while it heavily relies on sub-pixel scale displacement of a bulky camera. Based on the symmetric transformation of quadratic-phase metasurface, we propose scaled transverse translation (STT) utilizing planar optical elements (POEs) to facilitate sub-pixel sampling and remote super-resolution imaging. The STT module composed of a pair of planar optical elements with conjugated quadratic phase profile is fabricated and experimentally verified. By displacing POE within a millimeter-level range, we achieve sub-micron in imaging shift accuracy. Furthermore, the results of SR and SR enhanced Fourier ptychography imaging demonstrate significant compatibility and effectiveness of this module. The resolution improvement in FP imaging increases from 2× to 2.8× by sub-pixel sampling using this module. Moreover, defect reduction and contrast enhancement are obtained. With its advantages of light-weight, simple structure and ease of implementation, this method shows considerable potential for numerous imaging applications.

## 1 Introduction

High-resolution imaging is a long-standing pursuit in various scenarios including astronomy, remote sensing, and geological exploration. However, in long-range imaging, targets typically occupy only a few pixels, which complicates the capture of detailed features such as shape, size, and texture. The primary limitations on imaging resolution are attributed to both the restricted aperture size and pixel size corresponding to the Nyquist sampling frequency [1]. During the past few decades, the optical aperture has consistently increased, with notable examples including the Hubble space telescope and James Webb space telescope. Considering the tremendous efforts and costs associated with constructing large-aperture telescopic lenses, one of the most common methods to improve resolution involves increasing the number of pixels per unit area by reducing their physical dimensions. Nevertheless, this reduction in pixel size leads to a significant decrease in received luminous flux and signal-to-noise ratio (SNR), ultimately degrading image quality. Furthermore, the minimization of pixel also faces a great challenge in manufacturing techniques and readout circuits of detector arrays. Therefore, there exists a lower limit on pixel size for focal plane array.

Alternatively, algorithmic-based approaches are usually preferred over hardware-based solutions. Super-resolution (SR), the process of obtaining one or more high-resolution images from one or more low-resolution (LR) observations, has been a very attractive research topic over the past three decades [1]. Its practical applications span various fields including satellite and aerial imaging, medical image processing, facial image analysis, and biometrics recognition, to name a few. Various sub-pixel sampling techniques have been implemented to improve the imaging resolution by utilizing sub-pixel displacements in the imaging system generally achieved by shifting the illumination source [2], camera [3], [4], [5], mask [6], and/or sample [7] or micro-scanning techniques [8], [9], [10], [11], followed by a digital synthesis of a smaller effective pixel by merging these sub-pixel-shifted LR images. Although great advances have been achieved, the bulky volume of the moving camera and non-negligible inertia effect cause the difficulty in controlling image displacement accuracy at sub-micrometer scale. To address the deviations between the obtained multi-frame images, it is essential to perform motion estimation and registration between images [12], [13], and improving motion estimation accuracy has become one of the key focuses in traditional SR reconstruction efforts. Notably, the overall improvement in resolution has been modest; practical implementations achieve no more than a 2× improvement [14]. Although 4× improvement in resolution has been realized in some studies [15], these approaches do not guarantee fidelity due to the fact that spatial high-frequency components cannot be recovered [16]. Recently, one of the active sub-diffraction imaging techniques, known as the Fourier ptychography (FP), have been proposed, which also requires scanning the camera to different positions to collect multiple frame images. Distinctly, attributed to the principle of digitally coherent synthetic aperture, FP [16], [17], [18], [19], [20], [21] can realize significant resolution enhancements beyond those achieved by traditional SR techniques, which potentially allow for 10× improvements in resolution. With optimization, the working distance of FP imaging has been extand to over 100 m [22].

In recent years, metasurfaces have garnered extensive research interest due to their high integration, compact size, and multifunctionality, which can flexibly manipulate the amplitude, phase, and polarization of incident waves by tailoring the morphology of each meta-atom [23], [24], [25], [26], and modern optics has undergone a revolution known as meta-optics [27], [28] and Engineering Optics 2.0 (EO 2.0) [29], [30]. Inspired by the metasurface-assisted generalized refractive and reflective law [31], [32], symmetric [33], [34] and asymmetric spin–orbit interaction [35], [36], generalized geometric phase [37], [38], various novel design and functionalities have been developed, including vortex beam generation and sorting [39], [40], [41], wide-angle scanning and imaging [42], [43], [44], multi-channel nanoprinting and holography [45], [46], [47], [48], [49], [50], optical encryption [51], [52], optical neural network [53], among others [54], [55], [56], [57]. In particular, the cascaded metasurfaces offer greater degrees of freedom in harnessing the beam deflection [58], focusing point control [59], and vector light generation [60] via special optical field transformation. Consequently, it is anticipated to realize flexible and high-resolution sub-pixel sampling via cascaded metasurfaces with released requirements on displacement accuracy.

Inspired by the symmetric transformation of quadratic-phase metasurfaces [39], [42], we propose a scaled transverse translation (STT) by cascading a pair of planar optical elements (POEs) with conjugated quadratic phase profile. Akin to the well-known “twisted Moiré effect” in metasurfaces [61], [62], [63], the micrometer-scale high-resolution sub-pixel displacement can be easily realized by a millimeter-scale displacement between the cascaded POEs via STT. Owing to the parallel location between the cascaded POEs, this unique translation can be dubbed as “parallel Moiré effect”. We first present a generalized model of the STT and the phase profile of POEs. Then, we apply this proposed methodology for multiple-frame sub-pixel sampling of active imaging with fabricated POEs for SR imaging. Finally, we digitally synthesize the obtained SR images coherently to surpass the diffraction limit of the aperture size for remote sub-diffraction imaging.

## 2 Principles and methods

For photographic or telescopic imaging, a deviation in the angle of incoming light onto the lens results in an image shift on the focal plane. In accordance with the thin lens approximation model, the image conjugated with objects at infinity distance will shift at distance of *h* proportionally to the lens’s focal length *f*_*l*_ and the angle of light deviation *θ*, which can be expressed as

(1) $$h=f_{l}\tan\left(\theta \right)$$

Thus, changing the angle of incoming light *θ* can induce an image shift.

According to the symmetric transformation associated with quadratic-phase metasurfaces, a shift in the focal plane *s* leads to a change in output angle *θ*, which is proportional to the focal length *f*_*m*_ of the quadratic-phase POE, and this relationship is can be articulated as

(2) $$\theta ={\mathrm{si}n}^{-1}\left(f_{m}/s\right)$$

Consequently, if a focal vertical shift is created in front of the quadratic-phase POE adjacent to the imaging lens, the image shift *h* will occur, and the relationship between these two shifts can be expressed by combining Equations (1) and (2), yielding

(3) $$h=f_{l}\tan\left(\sin^{-1}\left(f_{m}/s\right)\right)$$

When the image shift is slight, the formula can be simplified as

(4) $$h=s\left(f_{l}/f_{m}\right)$$

It becomes evident that the ratio of the focal length, the ratio of the lens and the quadratic-phase POE determines the ratio of the displacement. By adjusting the appropriate ratio, precise control of the image displacement, referred to here as STT, can be achieved.

**Figure 1:**
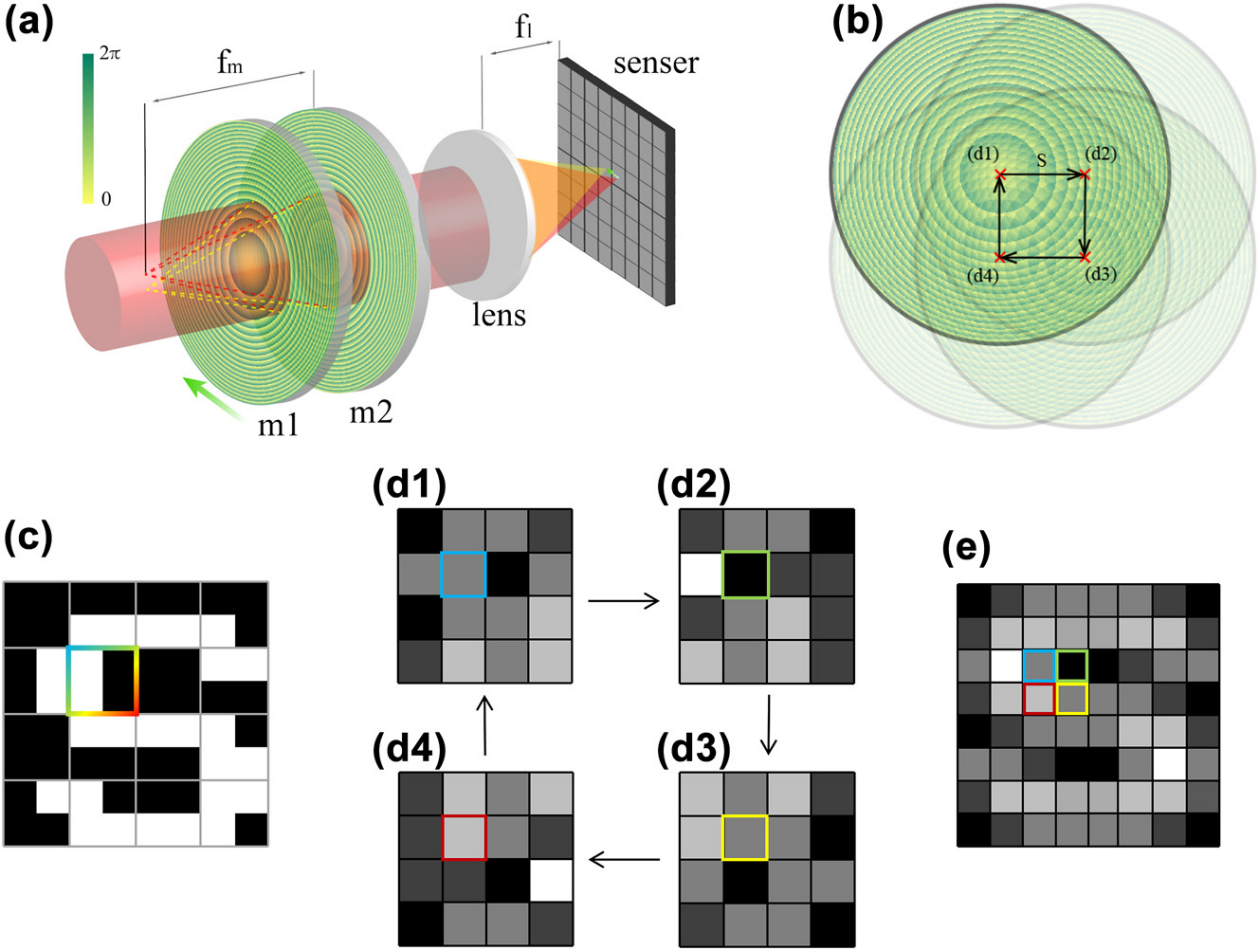
Principle of STT via a pair of POEs (m1 and m2) with conjugated quadratic phase profile. (a) The simplified structure and working principle. (b) The moving steps of the negative POE_2_. (c) The object to imaging with the target information scales below one pixel and (d1–d4) are the single shot imaging results to (c) according to the steps labeled in (b). (e) Is the sub-pixel up-sampled image.

The key issue of STT lies in achieving the focal shift in front of the quadratic-phase POE. A POE with a negative focal length in front of the quadratic-phase POE can fulfil this specific function. This negative POE, which is also represented by the quadratic phase, enables the collimated incident light beam to generate a virtual focus in front of the positive quadratic-phase POE. The movement of the virtual focus can be produced by adjusting the negative POE, and the displacement of the virtual focus can produce a beam deflection after passing through the positive POE, thus achieving a shift of the image plane. The fundamental principle underlying this mechanism is illustrated in Figure 1a, m1 represents the positive quadratic-phase POE, and m2 denotes the negative one. The ratio of the displacement of the negative lens to the displacement of the image is the same as the one derived earlier.

To achieve micron-level movement of the image (sub-pixel level) using moderate displacement of a negative POE, the focal length of quadratic phase POE needs to be much larger than the imaging lens. For traditional lenses, the surface sag variation is too small to fabricate with high accuracy. The high degree of design freedom and high phase control precision in phase modulation of the POEs make it advantageous in such scenarios. Additionally, the flatness and lightweight characteristics of POEs facilitate ease of movement.

In contrast to the traditional imaging system, sub-pixel imaging systems operate based on scanning techniques. For instance, a 2-times sub-pixel sampling image need four shots, with the graph on the image plane shifted four times, as shown in Figure 1b. Due to the resolution limit of the sensor, the image will be significantly blurred (Figure 1d1–d4). As we fuse the four low-res images along the opposite shifting direction, the high-resolution image has a greater likelihood of more closely resembling the object (Figure 1e). To get better reconstruction results, super-resolution imaging algorithms based on spatial complementary information between images predominantly rely on spatial domain constraints could be used. These methods establish a degradation process between low-resolution and high-resolution images, and through mathematical computation and the selection of constraint models, the high-resolution images are reconstructed via inverse problem-solving. The primary reconstruction methods include maximum posteriori (MAP) [64], [65], projection onto convex sets (POCS) [66], [67], and iterative back-projection (IBP) [68], [69]. We used a direct up-sampling algorithm here, it may not yield the best performance in resolution advance, but it can restore the high-resolution image in the rapidest way.

## 3 Results and discussions

### 3.1 Beam deflection using POEs based STT module

**Figure 2:**
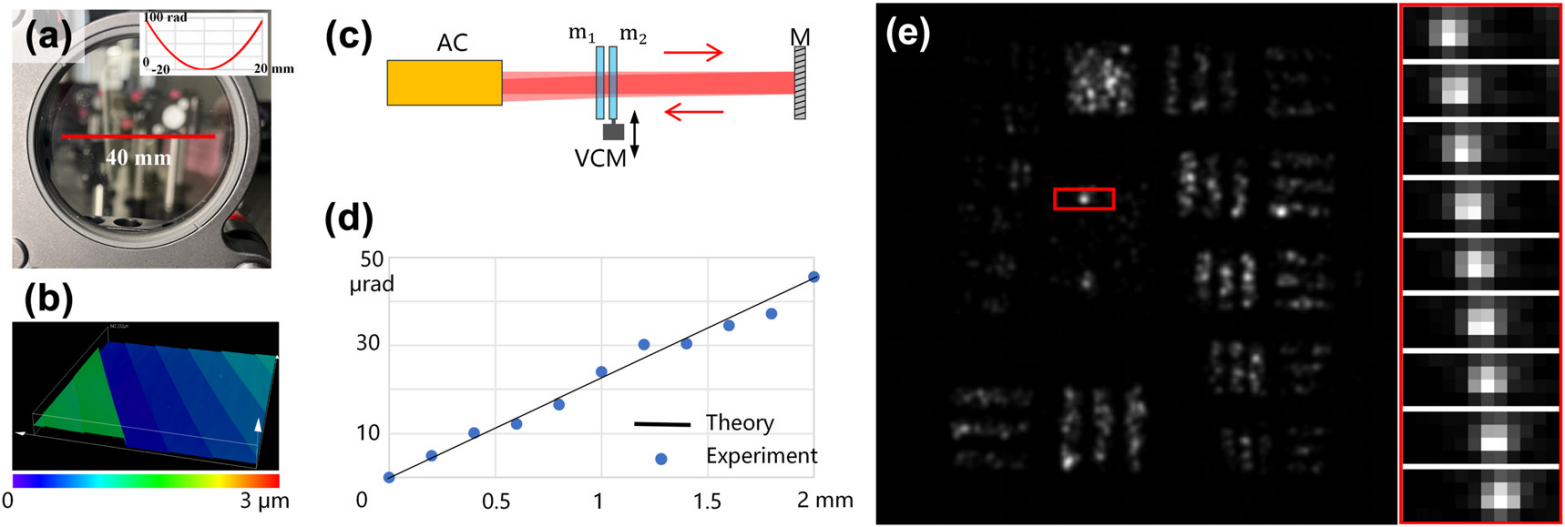
Characterization and test of the POEs module. (a) Picture of the POE. Insert curve shows phase profile along radial direction. (b) Surface feature of the POE. (c) The test system for light deflection. AC: auto-collimator, M: mirror, VCM: voice coil motor. (d) The test result of beam deflection caused by the module. (e) The sub-pixel image displacement caused by the POEs.

To verify the availability of the methods, we designed a pair of quadratic-phase POEs with focal length of approximately 25 m and −25 m at wavelength of 532 nm. Then, according to the phase distribution and central working wavelength, we obtained the structures of these two POEs and fabricated them using laser direct writing. The substrate applied was fused silica, with an aperture size of 40 mm. The structure of POE is fabricated as 8-stage diffraction optical element (DOE). The surface characteristics of fabricated samples are exhibited in Figure 2. The minimal width of the structure is 84 μm, while its depth is 149 nm. The fabrication process of POE with structure like this is mature. Additionally, the shift of images is only dependent to movement of one POE instead of the original position between two POEs. With large focal length, the mounting requirement of POEs is low. The module is easy to establish.

We evaluated the capability of the POE pair to deflect light beams. To measure the beam deflection with high accuracy during movement, we built a test system comprising an auto-collimator, a pair of POE under examination, a voice coil motor (VCM) to move one of the POEs and a mirror, as shown in Figure 2c. The auto-collimator emitted a collimating light that passed through the negative POE mounted on a VCM and a fixed positive POE. The light was then reflected by the mirror and passed through these two POEs again. The deflection of the returned light was measured by the auto-collimator. Since light traversed the module twice, any deflective angle reported by the auto-collimator should be divided by two for accurate representation. We conducted tests on the deflection with a movement step of 0.2 mm. The deflective angle created by the module is shown in Figure 2d. Results indicate that the beam deflection is linear and conforms to design expectations. Specifically, the deviation to the theoretical value is 1.15 ± 2.05 μrad, leading to an image displacement accuracy of 86 ± 154 nm for an imaging lens with a focal length of 75 mm, thus validating our module. For a detector with pixel size larger than 5 μm in our setup, the accuracy is high enough.

Subsequently, we integrated this STT module into an imaging system. The positive POE was fixed in front of the photographic lens with the negative one set close to it. The negative one was driven by VCM to produce precise dislocation in a rapid way. We altered the dislocation by 0.5 mm each time, corresponding to the half-pixel image displacement presented in Figure 2e, as we expected.

### 3.2 Sub-pixel imaging results via STT module

To match the wavelength of our applications, we redesigned a set of phase planes for an imaging lens with a focal length of 75 mm and an aperture of 18.75 mm (F/4). The sensor used in this system had a resolution of 1,280 × 1,024 pixels with each pixel size measuring 5 μm. We set the quadratic coefficient at 19.684 rad/(mm)^2^ for an operating wavelength of 1,064 nm, so a 2.5 μm image displacement might occur due to a dislocation of 0.5 mm between two POEs. The target was a USAF 1951 resolution chart, with a ceramic diffuse reflection surface, positioned about 1.9 m away from the lens.

**Figure 3:**
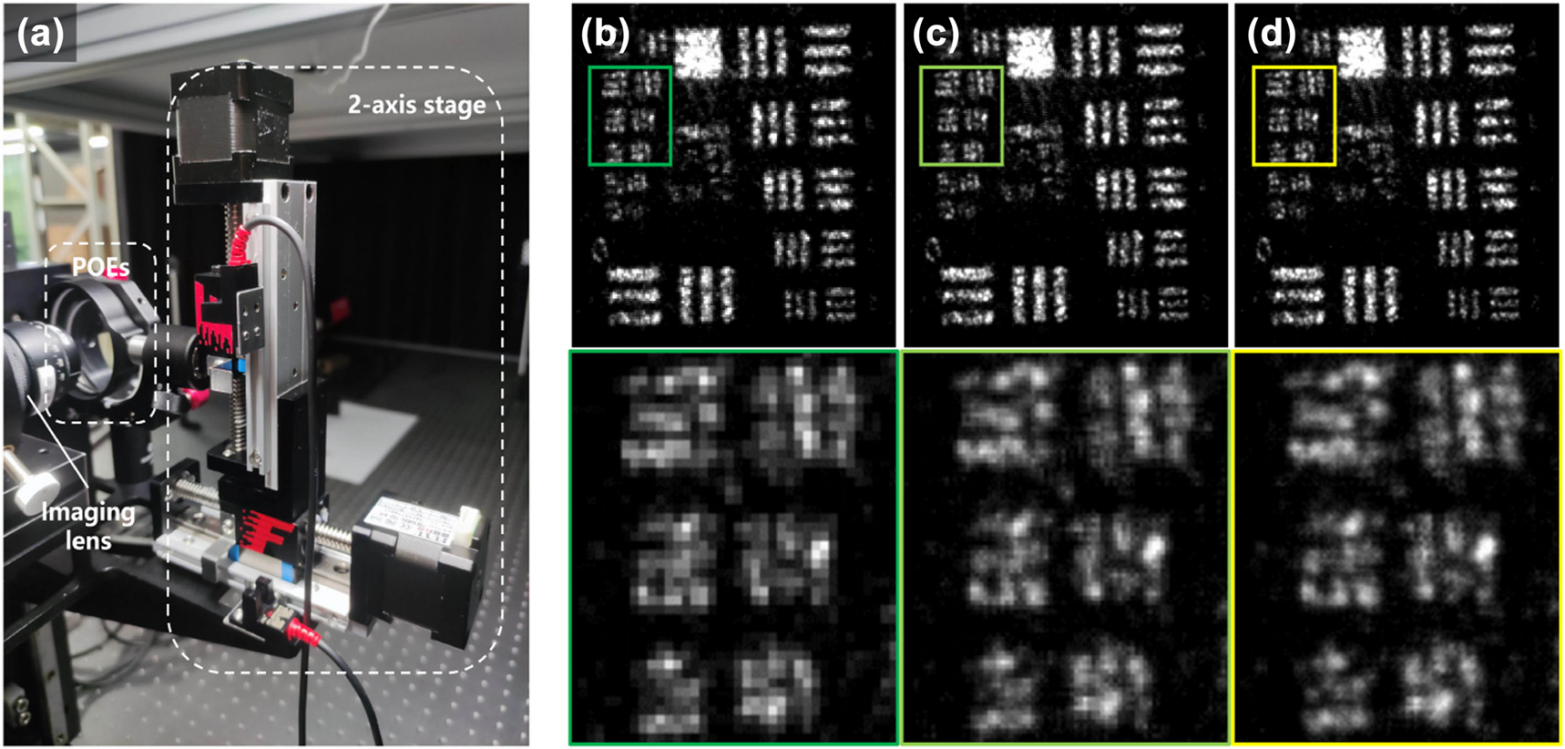
The results of sub-pixel imaging. (a) Two-dimensional sub-pixel imaging system. (b) Imaging result of single shot. (c) 2 times sub-pixel imaging result. (c) 4 times sub-pixel imaging result. The zoomed details are placed on the bottom.

The negative POE was mounted on a XZ-axis translation stage, as shown in Figure 3a. Using the method mentioned above, it was evident that the 2 times sub-pixel sampling image shown in Figure 3c had better resolution and contrast compared to the single shot image shown in Figure 3b. We also reconstructed a 4 times sub-pixel image by adjusting the movement step to 0.25 mm. The results are depicted in Figure 3d, demonstrating limited resolution improvement over the 2 times sampling sub-pixel image. It appeared that our method encountered challenges in high times sub-pixel image restoration. One possible reason was that the hidden high frequency information was hard to extract using this algorithm. Another reason could be that the optical cut-off frequency was limited by the photographic lens under these conditions.

### 3.3 Sub-pixel enhanced FP imaging

**Figure 4:**
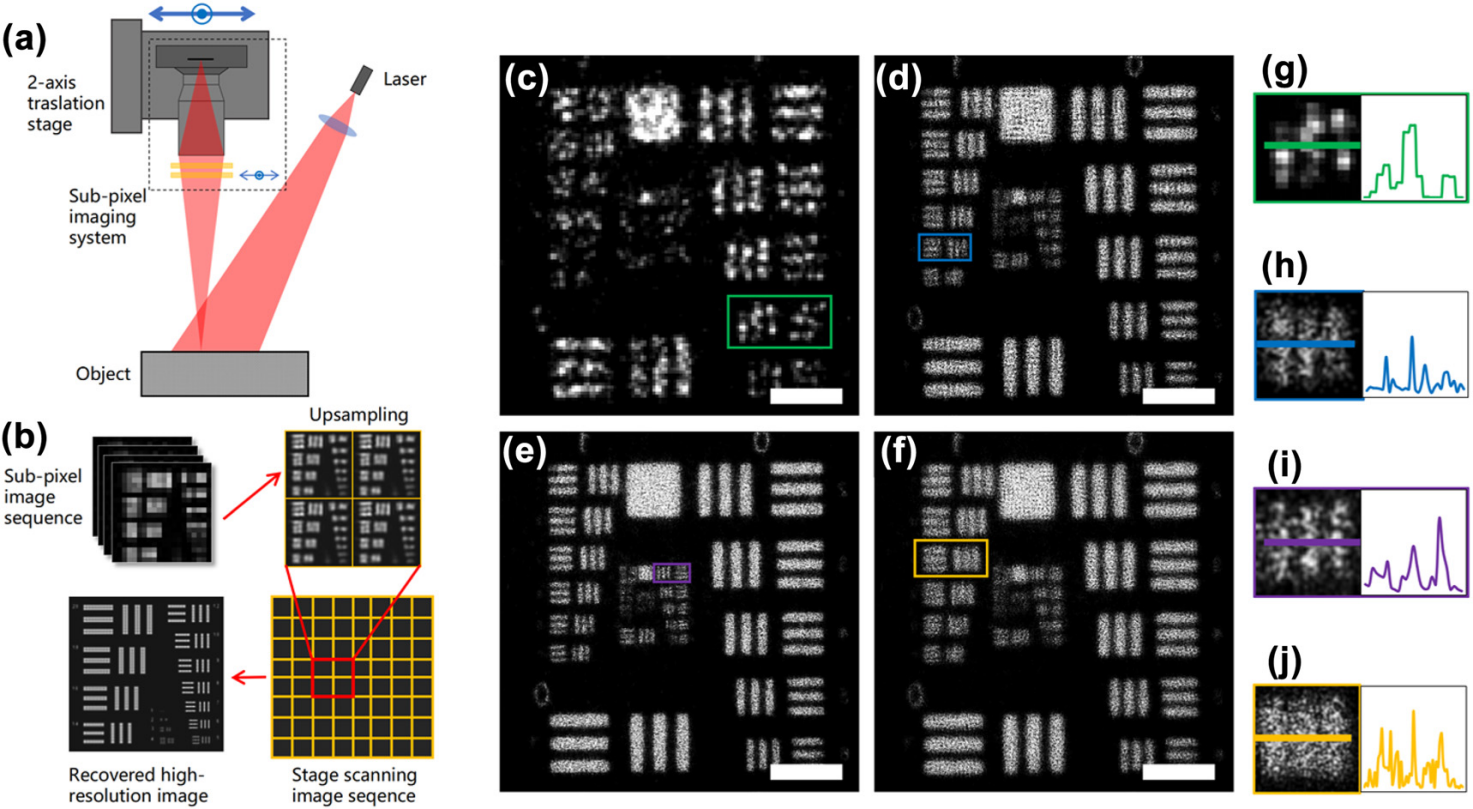
The resolution chart results of sub-pixel imaging in FP imaging. (a) Is the sub-pixel FP imaging system. (b) Is the super-resolution imaging and reconstruction process based on up-sampling module. (c) Is the single shot image. (d) Is FP recovered image. (e) Is 2 times sub-pixel FP recovered image. (f) Is 2 times interpolation FP recovered image. The profiles of the smallest distinguishable line-pair are shown in insert images. (g–j) are the enlarged images and intensity profiles of (c–f). Scale bar in (c–f): 3 mm.

As a novel approach to enhance the optical resolution, FP imaging required high sampling density on the image plane. Although the optical resolution got highly improved, the sampling density does not keep the pace, even with the up-sampling effect of the FP algorithm. To demonstrate substantial enhancement in resolution and contrast, we implemented the STT module in a FP system as presented in Figure 4a.

A diaphragm was implanted in front of the image lens to adapt the aperture to 12 mm for FP imaging. This image system with the sub-pixel module was then mounted onto a two-axis translation stage. The scheme of FP imaging system is shown in Figure 4a. The scanning program made the system move to form a 21 × 21 array with the step of 2.4 mm, coming up with a 60 mm synthetic aperture, which was 5 times of the single aperture. At each place of the 21 × 21 array, sub-pixel imaging was applied with the identical procedure described in the previous section, resulting in sub-pixel images with twice the sampling density. After captured all up-sampling images, an iterative algorithm to recover the high-resolution images was applied to obtain the final results, which is shown in Figure 4b.

We define the resolution based on the peak-valley contrast threshold set at 0.5. The contrast is defined as

(5) $$C=\frac{w-b}{w}$$

where w is the peak value of the line-pair and b represents the valley value. Figure 4c is the low-resolution image of a single shot, reaching the line-pair (0,5) resolution with the line width measuring 315.0 μm. Due to the under-sampling, the original FP image (Figure 4d) of 5 times synthetic aperture can only distinguish the line-pair (1,5) with the line width of 157.5 μm, just twice the resolution of the single aperture image.

In Figure 4e, the FP result from 2 times up-sampled images by our sub-pixel module shows higher resolution than the standard FP result. The resolution of line-pair (2,2) with the line width of 111.4 μm indicates a 2.8 times improvement than single aperture. In comparison, we resized the original images to double the size by bilinear interpolation instead of using actual sub-pixel module; then we put them into the FP recovering algorithm. The recovered result displays worse resolution in Figure 4f, for the interpolated intensities may be incorrect. It demonstrates the effectiveness of the sub-pixel module in FP imaging system.

**Figure 5:**
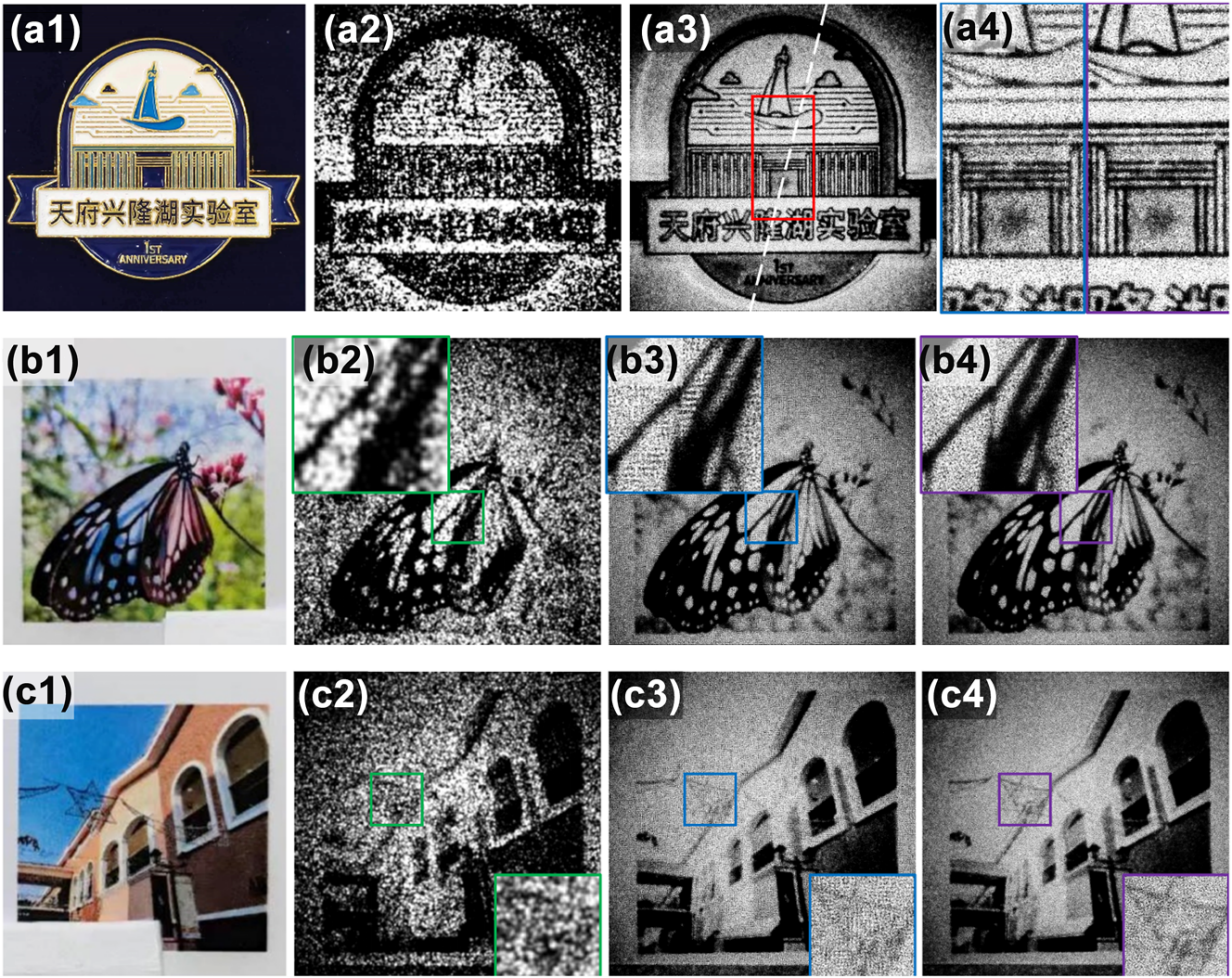
The results of physical object and printed pictures of sub-pixel FP imaging. (a1) A badge to be imaged. (a2) Single aperture image. (a3) Comparison between the FP recovered image (on the left) and sub-pixel FP recovered image (on the right). (a4) Zoomed details of the two images in (a3). (b1–b4) and (c1–c4) are the printed picture results, while (b2) and (c2) are the single shots; (b3) and (c3) are the FP recovered results; (b4) and (c4) are the sub-pixel FP results.

We also tested this system with a badge and some printed pictures. As it can be observed in Figure 5, the sub-pixel FP imaging system is capable of recovering higher resolution than the standard FP system. In the zoomed detail pictures, sub-pixel FP image system exhibits better line recovery performance (Figure 5a4) and lower constructed defects (Figure 5b3 and b4), resulting in an improved contrast ratio (Figure 5c3 and c4).

## 4 Conclusion and discussion

Based on the symmetric transformation of quadratic-phase metasurfaces, we proposed a methodology referred to as STT, which was implemented by cascading a pair of POEs with conjugated quadratic phase profiles. With proper phase design, high accuracy image displacement could be achieved by moderate motion of the POEs. Subsequently, this proposed methodology was applied for multiple-frame sub-pixel sampling of active imaging with fabricated POEs for SR imaging. Higher resolution of single-aperture imaging results and synthetic aperture recovered results demonstrated the effectiveness of this method. With advantages of lightweight and simple structure, this approach can be integrated to any other imaging system for sub-pixel imaging or image stabilization, with minimal modifications required. It shows significant potential across diverse imaging applications.

The performance of this method could be improved further. As the POE is diffractive element, the focal length of POE is inversely proportional to wavelength, described as ${\text{f}}_{\lambda }=\left(\lambda_{0}/\lambda \right){\text{f}}_{\lambda_{0}}$ . It will change the ratio between POE shift and image shift. When the bandwidth is too wide, the images with different wavelengths will separate, introduce a non-negligible image blur. Meanwhile, the diffractive efficiency of POE is also related to wavelength. In this work, the active illumination imaging system with narrow bandwidth avoids this effect. To applied these methods to wide bandwidth application, POE based on harmonic diffraction could retard this problem.
